# Diagnosis and management of intestinal Behçet’s disease

**DOI:** 10.1007/s12328-014-0488-0

**Published:** 2014-04-20

**Authors:** Tadakazu Hisamatsu, Makoto Naganuma, Katsuyoshi Matsuoka, Takanori Kanai

**Affiliations:** 1 Division of Gastroenterology and Hepatology, Department of Internal Medicine, School of Medicine, Keio University, 35 Shinanomachi, Shinjuku-ku, Tokyo, 160-8582 Japan; 2 Center for Diagnostic and Therapeutic Endoscopy, School of Medicine, Keio University, Tokyo, Japan

**Keywords:** Intestinal Behçet’s disease, Anti-TNFα mAb, Trisomy 8, Adalimumab

## Abstract

Behçet’s disease (BD) is a chronic relapsing disease with multiple organ system involvement characterized clinically by oral and genital aphthae, cutaneous lesions, and ophthalmological, neurological, and/or gastrointestinal manifestations. Little clinical evidence is available regarding the management of patients with intestinal BD, despite recognition that the presence of intestinal lesions is a poor prognostic factor, causing perforation and massive bleeding. Many recent case reports have suggested that anti-tumor necrosis factor alpha (TNF)α monoclonal antibodies (mAbs) are effective in patients with intestinal BD. Adalimumab, a fully human anti-TNFα mAb, has been approved in Japan for the treatment of intestinal BD. Here, we review the pathogenesis, diagnosis and management of intestinal BD, including evidence of the efficacy of anti-TNFα mAbs.

## Introduction

Behçet’s disease (BD) was first defined in 1937 by Hulusi Behçet [1], a Turkish dermatologist, as a triad of recurrent aphthous stomatitis, genital aphthae and relapsing uveitis. This disease is highly prevalent along the Silk Road, including Japan, Korea, the Middle East, and the Mediterranean region.

Although intestinal lesions associated with BD may cause serious complications, such as perforation, and decreasd quality of life, the diagnosis and management of intestinal BD lesions has not been standardized. Empirical therapies have been used anecdotally to treat intestinal BD. In Japan, adalimumab (ADA), an anti-tumor necrosis factor alpha (TNFα) monoclonal antibody (mAb), was approved for the treatment of intestinal BD in 2013. The introduction of anti-TNFα mAbs has altered treatment strategies and may improve the long-term prognosis of patients with intestinal BD. Here, we review current topics in intestinal BD, including its clinical characteristics, diagnosis, and management.

## Diagnosis of intestinal Behçet’s disease

BD is regarded as a chronic relapsing disease with multiple organ system involvement characterized clinically by oral and genital aphthae, cutaneous lesions, and ophthalmological, neurological, and/or gastrointestinal manifestations [2, 3]. Several diagnostic criteria for BD have been proposed. The widely used International Study Group (ISG) for Behçet’s disease criteria include recurrent oral ulcer, plus at least two of the following four factors—recurrent genital ulcers, eye lesions, skin lesions, and positive pathergy test [4]. The Japanese criteria proposed in 2004 are also widely used [5].

**Fig. 1:**
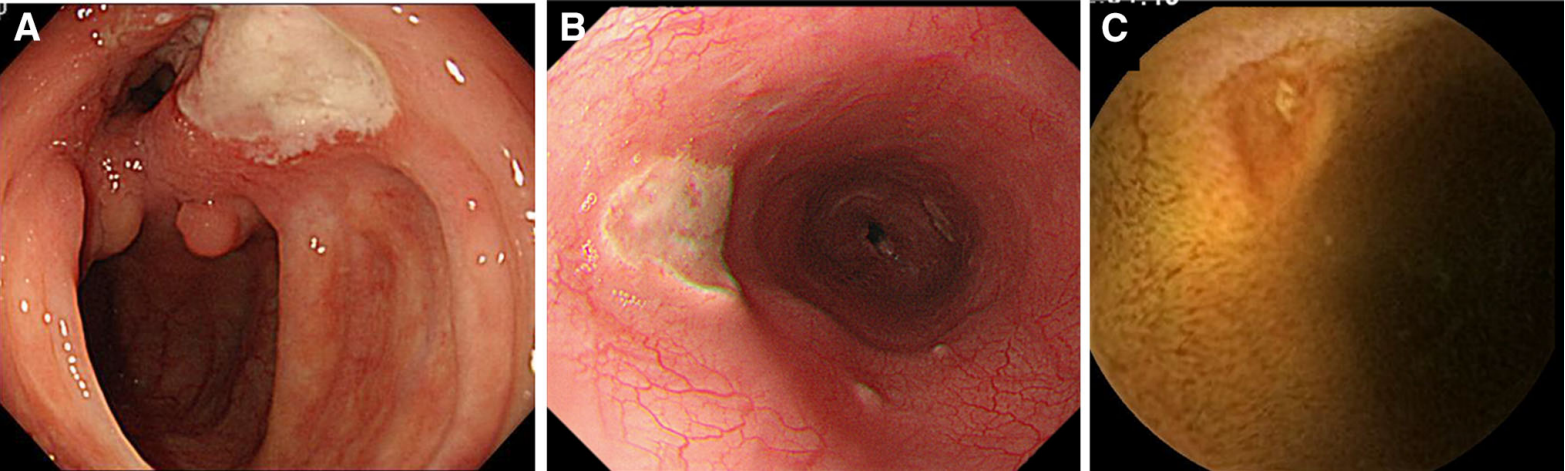
Gastrointestinal lesions in BD. **a** A typical giant *oval-shaped* deep punched-out ulcer in the ileocecal area. **b** An atypical *oval-shaped* ulcer in the *middle* part of the esophagus in a patient with intestinal BD. **c** A discrete ulcer in the small intestine detected by capsule endoscopy in a MDS patient associated with trisomy 8

Approximately 3–16 % of patients with BD have gastrointestinal tract involvement [6]. A retrospective analysis of 2,313 patients with BD found that the male/female patient ratio was 1.03, with gastrointestinal involvement present in 1.4 % of both males and females [7]. A typical gastrointestinal lesion consists of a giant oval-shaped deep punched-out ulcer in the ileocecal area (Fig. 1a); however, involvement of the esophagus and small intestine has also been reported. The most common gastrointestinal symptoms are abdominal pain, diarrhea, and bleeding. Deep ulcers are responsible for the most common intestinal complications, such as severe bleeding and perforation. Therefore, intestinal lesions have been considered a factor associated with poor prognosis in BD patients, resulting in emergency abdominal surgery and bowel resection [8].

Confusion has arisen regarding the terminology used to describe this condition. Among the terms used are ‘intestinal BD’, ‘entero-BD’, and ‘intestinal lesions associated with BD’, with the various terms possibly due to a lack of standardized diagnostic criteria. In this review, we use the term ‘intestinal BD’ according to the diagnostic criteria reported by Kobayashi et al. [9]. Briefly, intestinal BD is diagnosed in patients meeting the Japanese diagnostic criteria of BD [5], by the presence of a typical oval-shaped large ulcer in the ileocecum. However, we have often encountered patients with these ulcers in the ileocecum who do not have typical BD manifestations. These patients, who cannot be diagnosed with intestinal BD by Japanese criteria, have been described as having ‘simple ulcer syndrome’ [10]. To date, similarities and differences in the pathogenesis, histopathology, and prognosis of Japanese patients with intestinal BD and simple ulcer syndrome have not been identified, although neutrophilic phlebitis may be involved in the pathogenesis of both [11]. The clinical manifestations of BD often show spatial and temporal diversity, making it difficult to differentiate between intestinal BD and simple ulcer syndrome in some patients. In addition, we often encounter patients with BD and atypical gastrointestinal lesions. Again, similarities and differences in the pathogenesis of these atypical lesions and typical oval-shaped ulcers have not been identified. A Korean group proposed novel diagnostic criteria for intestinal BD in Korean patients with ileocolonic ulcers [12]. They suggested that systemic BD patients with typical ileocecal ulcers should be diagnosed as having ‘definite intestinal BD’, patients with typical ileocecal ulcer and oral ulcers and patients with systemic BD and atypical ulcers should be diagnosed as having ‘probable intestinal BD’, and patients with typical ileocecal ulcers without any BD symptoms should be diagnosed with ‘suspected intestinal BD’.

Although an oval-shaped ulcer at the ileocecum is considered typical of intestinal BD, esophageal lesions have also been reportedly associated with BD [13–17] (Fig. 1b). For example, one study reported that the incidence of esophageal involvement was relatively low (11 %) [18], and a retrospective analysis of 842 Korean patients diagnosed with BD found that 129 (15.3 %) experienced upper gastrointestinal symptoms, but esophageal involvement was found in only six (4.7 %) of these 129 patients [19]. Esophageal lesions may be helpful in the diagnosis of intestinal BD, but the necessity of upper gastrointestinal examination in asymptomatic BD patients has not been determined.

## Differential diagnosis of intestinal BD

**Fig. 2:**
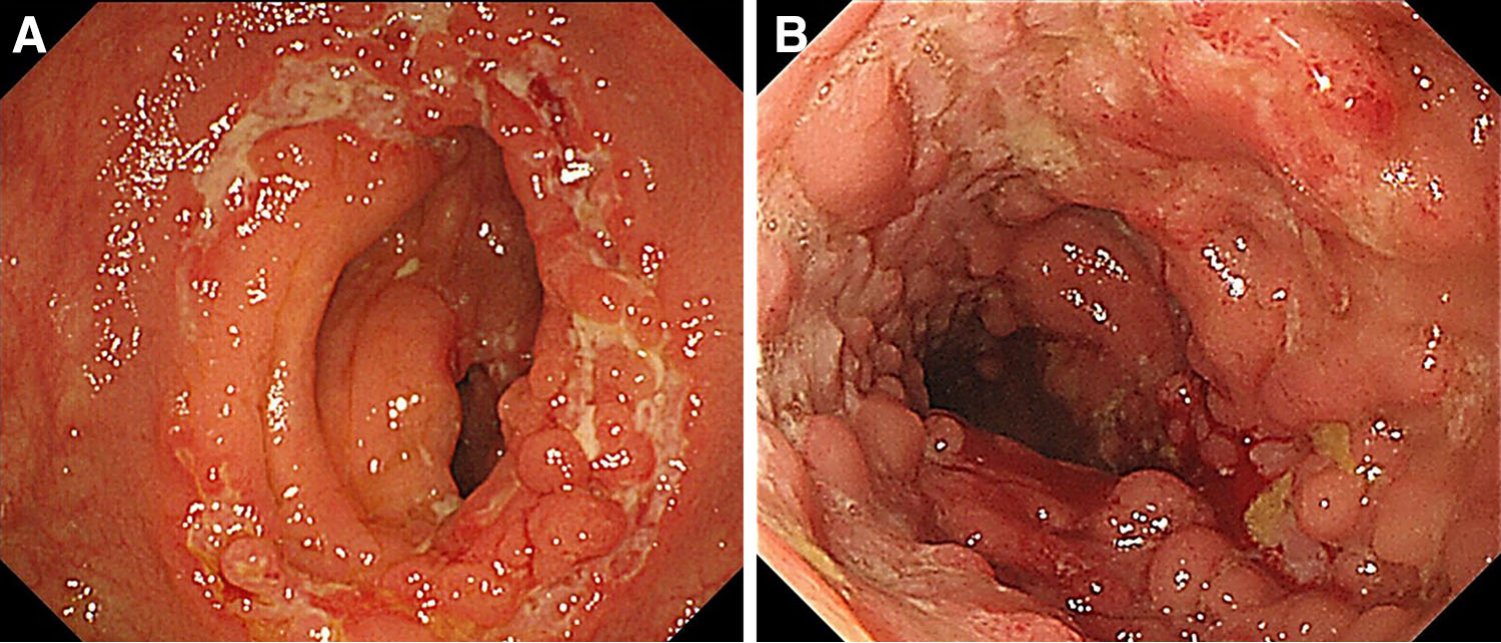
Differential diagnosis of intestinal BD. **a** Annular ulcers in patients with active TB. **b** Longitudinal ulcers and a cobblestone appearance in a patient with CD

Intestinal tuberculosis (TB), Crohn’s disease (CD), and other diseases with intestinal ulceration should be excluded. Ruling out intestinal TB is especially important, because the immunosuppressive therapy used to treat BD, including corticosteroids and anti-TNFα mAbs, can exacerbate intestinal TB. Methods of diagnosing intestinal TB include tissue culture, tissue PCR and interferon-gamma release assays (IGRA), in addition to general examinations such as chest X-ray and tuberculin test. Endoscopic findings of intestinal TB often include annular ulcer and scarred areas with discoloration (Fig. 2a).

The differential diagnosis between intestinal BD and CD is often difficult, since several extraintestinal manifestations, such as oral ulcers and arthralgia, are seen in both diseases. Typical endoscopic and radiological findings in patients with CD include longitudinal ulcers and a cobblestone appearance (Fig. 2b). Anal lesions are more common in CD than in intestinal BD. Balloon small intestinal endoscopy and capsule endoscopy have recently been reported to be useful for the diagnosis and monitoring of patients with intestinal BD [20–23] (Fig. 1c).

## Pathogenesis of intestinal BD

### Genetic factors

Few cases of familial intestinal BD have been reported to date, suggesting the contribution of genetic factors in its pathogenesis [24, 25]. Recently, genome-wide association studies (GWAS) have identified several genes associated with susceptibility to BD including the interleukin (IL)-23R, IL-10, STAT, and HLA-B51 genes [26–29]. However, few genetic factors associated with the phenotype of intestinal BD have been identified. The positive ratio of HLA-B51 has been reported to be lower in patients with intestinal BD associated with myelodysplastic syndrome (MDS) than in BD patients without intestinal involvement [18]. The number of copies of the *DEFA1* gene, which encodes α-defensin-1, has been reported to correlate with intestinal involvement in BD [30], and familial cases of BD with intestinal lesions have been reported to be associated with NEMO mutations [31].

### Immunological abnormalities

Susceptible genes identified by GWAS strongly suggest that abnormal immunological responses may play a role in the pathogenesis of BD. However, the precise mechanisms underlying the pathogenesis of intestinal BD have not yet been identified. Abnormal innate immune responses have been reported to be associated with intestinal BD [30, 32]. Moreover, tissue samples taken from intestinal lesions of BD have been found to express interferon gamma (IFNγ), TNFα and IL-12 mRNAs, indicating skewed Th1 responses [33]. Similarly, an investigation of cytokine expression in ileal biopsy specimens from patients with intestinal BD reported Th1 skewing [34]. Recent reports showing the efficacy of anti-TNFα mAb suggest the importance of TNFα in the pathogenesis of intestinal BD.

### Trisomy 8 and intestinal ulcers

Although BD and MDS are two different disease entities, some BD patients have bone marrow disorders such as MDS and aplastic anemia. MDS is a clonal hematologic disease with cytogenetic abnormalities. The most common chromosomal abnormality in BD patients with MDS is trisomy 8. A review of 62 Japanese patients with BD-associated MDS found that, among the 45 patients with abnormal karyotypes, 39 (86.7 %) had trisomy 8 [35]. Similarly, an analysis of the clinical features of 13 patients with BD and bone marrow disorders found that seven (54 %) had trisomy 8 [36]. Trisomy 8 may also be associated with the development of intestinal ulcers in patients with MDS [37]. The mechanisms by which trisomy 8 is associated with intestinal ulcers has not been determined, although autoimmune mechanisms play a role in the development of hematopoietic disorders such as MDS and aplastic anemia [38, 39]. Gene expression analysis of CD34^+^ hematopoietic cells in patients with trisomy 8 showed over-expression of proinflammatory cytokines [40]. In addition, trisomy 8 was associated with low copy numbers of the human beta-defensin 2 gene, which plays a role in human innate immunity [41]. Interestingly, a case report showed atypical endoscopic findings of intestinal ulcers in patients with BD and trisomy 8 [42], differing from the typical endoscopic findings of a giant oval punched-out ulcer at the ileocecum. Further investigations are needed to assess the similarities and differences between intestinal BD and intestinal ulcers in BD patients with trisomy 8.

## Management and therapy

### Conventional treatments and disease prognosis

Clinical evidence regarding the management of patients with intestinal BD is limited. Among the agents used empirically, 5-aminosalicylic acid (5-ASA), systemic corticosteroids, thalidomide, colchicine and immunosuppressive agents have been used. A study in Korea showed that 5-ASA/sulfasalazine therapy could maintain remission in patients with intestinal BD, although younger age (<35 years), higher C-reactive protein (CRP) level, and higher disease activity were associated with a poor response to 5-ASA/sulfasalazine [43]. Mesalazine was shown to have benefits in the treatment of esophageal ulcers in a patient with intestinal BD [44]. Corticosteroids are generally used to induce clinical remission in intestinal BD patients with moderate to severe activity [15, 45–47]. Immunosuppressants have also been used successfully. For example, a retrospective analysis of 272 patients with intestinal BD in a single center described the efficacy of thiopurine maintenance therapy. Of these 272 patients, 67 (24.6 %) received their first course of thiopurine therapy in the center, with 39 (58.2 %) of these 67 patients maintained on thiopurines. The cumulative 1-, 2-, 3- and 5-year relapse rates after remission were 5.8, 28.7, 43.7, and 51.7 %, respectively [48]. Methotrexate (MTX) has also been used to treat refractory intestinal BD [49]. Oral tacrolimus was effective in a patient with intestinal BD [50], and thalidomide, an agent with anti-inflammatory and immunomodulatory properties, has also been found to be effective [51–53].

In response to a request to standardize treatment of intestinal BD, the Japanese Inflammatory Bowel Disease Research Group, supported by the Japanese Ministry of Health, Labour and Welfare, proposed the first set of consensus statements in 2007 for the management of intestinal BD [9]. This consensus recommended systemic corticosteroids for induction therapy and thiopurines for refractory intestinal BD as standard therapies, with anti-TNFα mAb described as optional.

Despite reports showing the beneficial effect of medical therapies, patients with intestinal BD often require surgical treatment and may develop post-operative recurrence. Thus, intestinal BD, in at least a subpopulation of patients, should be considered a progressive disorder that causes disability, similar to CD. Since it is difficult to predict which patients will experience complicated disease courses, therapy should be individualized and depend on monitoring of individual patients. In our retrospective analysis of 20 patients, ocular and ileal lesions were risks for surgery [54]. Postoperative recurrence of intestinal ulcers was observed in seven of nine patients with intestinal BD who had undergone a total of 15 operations [55]. A retrospective analysis of 72 Korean patients with intestinal BD who underwent surgery showed that 42 (58.3 %) experienced recurrence after surgery, with 22 (30.6 %) requiring re-operations. The cumulative 2- and 5-year recurrence rates after surgery were 29.2 and 47.2 %, respectively [56]. A retrospective evaluation of 130 patients with intestinal BD during the first 5 years after diagnosis revealed five different clinical courses, with the most frequent being persistent remission or mild clinical activity (56.2 %) and only 16.2 % having a severe clinical course. Younger age, higher erythrocyte sedimentation rate (ESR), CRP concentration, and disease activity index, and lower albumin concentration at diagnosis were factors associated with poor patient prognosis [57].

### Anti-TNFα monoclonal antibodies

The efficacy of anti-TNFα mAbs in intestinal BD was first reported in 2001. Treatment with infliximab (IFX) of two patients with intestinal BD resistant to conventional therapy, including prednisolone, one with 3 mg/kg and the other with 5 mg/kg IFX, resulted in the rapid (within 10 days) reduction of intestinal lesions and extraintestinal manifestations [53]. Remission in both patients was maintained with thalidomide, not IFX. In addition, a patient with chronically active, steroid-dependent BD involving the gastrointestinal tract who was treated with four doses of IFX over a period of 6 months showed a reduction in CD activity index (CDAI) from 270 points before infusion to 13 points by week 2, with remission sustained despite the complete withdrawal of steroids [58]. Colonoscopy 10 weeks after the first infusion showed marked endoscopic and histological improvement. After these reports suggesting the rapid efficacy of IFX, several groups have assessed the efficacy of anti-TNFα in intestinal BD [59, 60]. For example, six Japanese patients with intestinal BD, all of whom were steroid dependent and refractory to other treatments, received IFX induction therapy (5 mg/kg at 0, 2, and 6 weeks), followed by maintenance therapy every 8 weeks [61]. Four of these six patients achieved and maintained remission with IFX. The other two patients, both of whom had ileal ulceration, required surgery, but one has maintained remission by IFX after surgery. A retrospective analysis of 28 patients with intestinal BD who received at least 1 dose of IFX and were followed-up for a median 29.5 months, resulted in response rates to IFX at 2, 4, 30, and 54 weeks of 75, 64.3, 50, and 39.1 %, respectively, and clinical remission rates of 32.1, 28.6, 46.2, and 39.1 %, respectively [62]. Multivariate analysis indicated that older age at diagnosis (≥40 years), female sex, longer disease duration (≥5 years), concomitant immunomodulator use, and achievement of remission at week 4 were predictive of sustained response. BD patients with intestinal lesions have a risk of multiple operations, but postoperative use of anti-TNFα has not been shown to reduce postoperative relapse rates and risk of multiple operations. IFX was used as rescue therapy for a patient with an unhealed anastomosis site and early recurrent ulcers after bowel resection [63]. IFX has also been reported effective in treating pediatric patients with intestinal BD, including a 15-year-old girl with refractory intestinal BD who responded rapidly to IFX [64] and a pediatric patient with progressive, refractory pediatric BD with intestinal lesions who responded to IFX [65].

Fewer reports have described the clinical efficacy of ADA. One patient with intestinal BD was treated with ADA monotherapy [66], whereas another was diagnosed with intestinal BD despite ADA treatment for underlying ankylosing spondylitis [67]. In Japan, a phase 3, non-randomized, non-controlled, one-arm, clinical trial tested ADA for intestinal BD [68]. Patients were given 160 mg ADA at week 0, 80 mg at week 2, and 40 mg every other week, beginning at week 4. The primary endpoint was ‘marked improvement’ rate at week 24, with ‘marked improvement’ defined according to the physicians’ global assessment of gastrointestinal symptoms and endoscopic improvement. The ‘marked improvement’ and complete remission rates at week 24 were 45 and 20 %, respectively. Based on the results of this clinical trial, ADA was approved in Japan to treat intestinal BD in May 2013. A clinical trial has also tested IFX for intestinal BD in Japan, and the second edition of consensus statements for the diagnosis and management of intestinal BD has proposed anti-TNFα mAb as a standard therapy for patients with moderate to severe intestinal BD [68].

### Can anti-TNFα mAb change therapeutic strategy of intestinal BD?

**Fig. 3:**
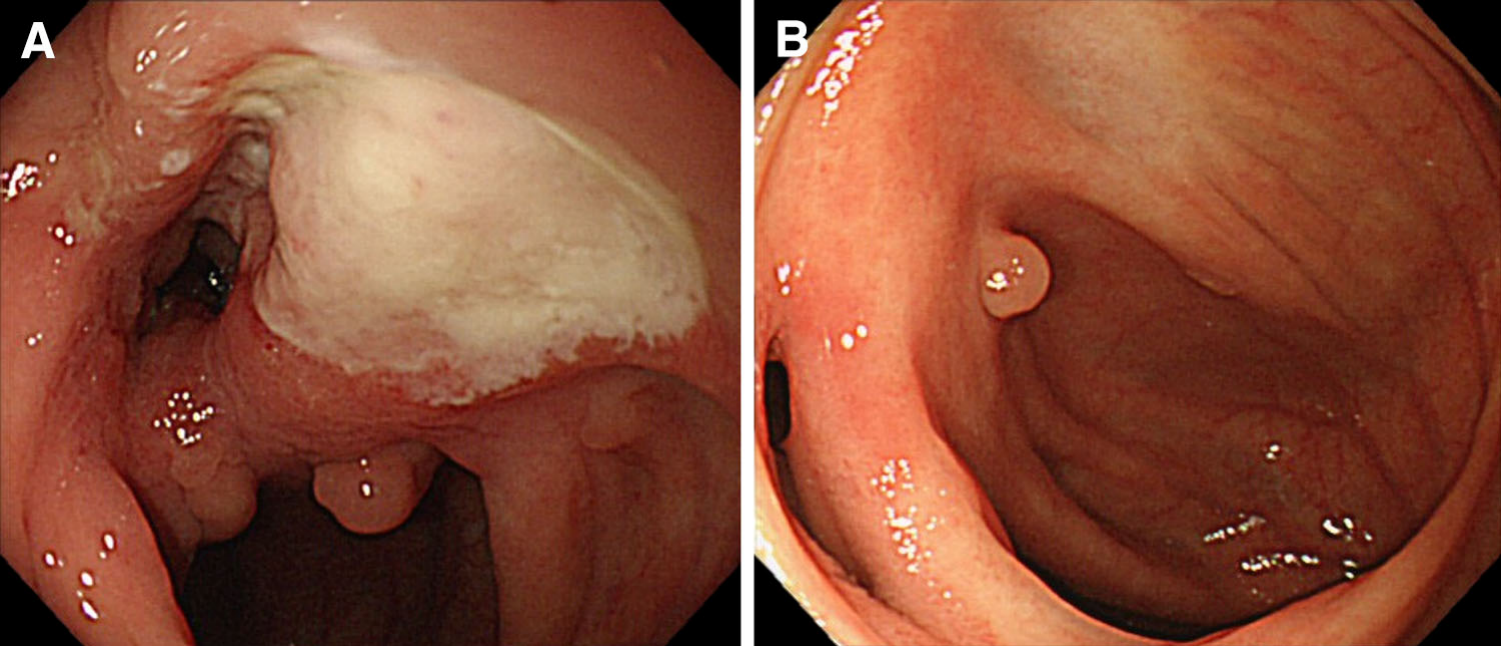
IFX treatment can induce ‘mucosal healing’ of intestinal BD lesions. **a** A typical giant *oval-shaped* ulcer observed before initiation of IFX. **b** Dramatic improvement of ileocecal lesions after treatment with IFX (the patient was reported by Maruyama et al. [72])

CD is regarded as a progressive disability of the digestive tract. Early intervention with anti-TNFα mAbs may alter the natural history of CD and improve the long-term prognosis of patients with this disorder [69]. Sub-types of BD are also progressive diseases, with BD uveitis causing loss of vision and intestinal BD requiring bowel resection. Thus, it is important to determine if anti-TNFα mAb treatment can improve the long-term prognosis of these patients. Although anti-TNFα mAb has been reported to reduce the risk of visual loss in patients with BD uveitis [70], its ability to reduce the risk of surgery in patients with intestinal BD has not been fully investigated. Since clinical symptoms and clinical activity index are often subjective in inflammatory bowel disease (IBD), discrepancies between clinical symptoms and endoscopic findings have been observed in IBD patients. Therefore, endoscopic findings are regarded as more important in evaluating the management of IBD patients. Mucosal healing, defined as endoscopic remission, has become the goal of IBD treatment to improve the long-term prognosis [71]. In contrast, there is no evidence indicating that mucosal healing should be a treatment target for improving the long-term prognosis of patients with intestinal BD, although the concept of ‘mucosal healing’ may be applicable in the management of these patients (Fig. 3a, b). For example, an analysis of 10 patients with intestinal BD who were treated with IFX and MTX reported that ileocecal ulcerations disappeared in nine of these patients (90 %) 12 months after initiation of IFX [49]. A patient with intestinal BD who was treated with IFX monotherapy successfully maintained clinical remission and complete mucosal healing for 6 years [72]. A retrospective analysis of the correlation between endoscopic parameters and clinical activity index in 167 patients with intestinal BD found that, although the number of intestinal ulcers and volcano-shaped ulcers were predictive of severe clinical index score, the correlation between endoscopic severity and clinical activity index was weak [73].

Thus, as in IBD, anti-TNFα mAb treatment may achieve mucosal healing and improve the long-term prognosis in patients with intestinal BD. To ensure the maximal efficacy of anti-TNFα mAb therapy, the concept of ‘early CD’ has been proposed. Subgroup analysis of the CHARM trial showed that ADA was superior to placebo in maintaining clinical remission in patients with moderately to severely active CD after 1 year of treatment, regardless of disease duration [74]. Clinical remission rates through 3 years of treatment were highest in the group with the shortest disease duration. However, the optimal timing of anti-TNFα mAb treatment in intestinal BD has not been determined. Despite anecdotal evidence showing the efficacy of combinations of immunomodulators with anti-TNFα mAbs as induction and maintenance treatment in intestinal BD, there is no consensus regarding their use. Even in CD, it remains unresolved whether anti-TNFα mAbs should be used in combination with immunomodulators [75–78]. Although one study reported the effectiveness of MTX plus IFX [49], another described a patient successfully maintained with IFX monotherapy [72].

## Conclusion

In reviewing the latest reports on the diagnosis and management of intestinal BD, we found that anti-TNFα mAb is a promising treatment for patients with this disorder. However, several issues remain to be resolved. Genomic analysis of patients with intestinal BD, as well as determining the mechanism of action of anti-TNFα mAbs, may provide insight into the pathogenesis of this disorder. Clinically, it is necessary to formulate global diagnostic criteria and an objective disease activity index. Treatment with anti-TNFα mAbs will likely alter disease prognosis, although these agents are not necessary in all patients with intestinal BD. Most importantly, it is necessary to identify high-risk patients and to monitor their disease activity.

## Abbreviations

ADA: Adalimumab
BD: Behçet’s disease
CDAI: Crohn’s disease activity index
CRP: C-reactive protein
5-ASA: 5-Aminosalicylic acid
IFX: Infliximab
mAb: Monoclonal antibody
MDS: Myelodysplastic syndrome
MTX: Methotrexate
TNF: Tumor necrosis factor
